# A Review of the Migration and Transformation of Microplastics in Inland Water Systems

**DOI:** 10.3390/ijerph19010148

**Published:** 2021-12-23

**Authors:** Yamei Cai, Chen Li, Yaqian Zhao

**Affiliations:** 1 Department of Municipal and Environmental Engineering, Faculty of Water Resources and Hydroelectric Engineering, Xi’an University of Technology, Xi’an 710048, China; 2180421304@stu.xaut.edu.cn (Y.C.); 2180420141@stu.xaut.edu.cn (C.L.); 2 State Key Laboratory of Eco-Hydraulics in Northwest Arid Region, Xi’an University of Technology, Xi’an 710048, China

**Keywords:** microplastics, sources, migration, transformation, inland water

## Abstract

Plastic productions continue to grow, and improper management of plastic wastes has raised increasing concerns. This reflects the need to explore the microplastics in water bodies. Microplastics have been regarded as emerging pollutants in water systems. In recent years, large numbers of studies across the world were conducted to investigate the distribution, behavior and the integrated impacts of microplastics in both the marine environment and the freshwater environment. Compared with the marine environment, the migration and transformation of microplastics in inland water systems seem more informative as they may reach the marine environment as one of their final destinations. Based on the updated literature, this review aims at overviewing the migration and transformation processes/behavior of microplastics in rivers, lakes and reservoirs. As for the migration, the microplastics’ fate is from manufacturing, consuming, discarding to migrating and returning to the human society which could form a closed though complicated circle. For transformation, microplastics experience five stages of their fate in inland water systems. These include changing into suspending pieces; ending up deposited as the sediment; resuspending under various changing conditions; ending up via burying into the soil as the part of the riverbed; reaching the marine environment; and being ingested by organisms and also becoming entangled with aquatic plants, etc. It is highly expected that this review can provide a valuable reference for better understanding microplastics’ migration and transformation mechanisms and a guide for the future study of microplastics in an inland water environment.

## 1. Introduction

Since plastic was first produced in 1907 [1], it has facilitated human’s production methods and lifestyles. Owing to its hard degradation property, the whole world has been confronted with the ‘White Pollution’ for decades, implying that the plastic products have been discarded carelessly, thereby polluting the surrounding environment. Microplastics were firstly referred to in ‘Lost at Sea: Where is all the plastic?’ in 2004 [2]. Since then, they have caused international attention especially in recent years. Originating from textiles, petroleum products, personal care and toiletries, microplastics usually refer to fragments of any type of plastic less than 5 mm in length [3]. They are further divided into primary microplastics (i.e., the plastic with a microscopic size typically in 2–5 mm from industrial productions) and secondary microplastics (i.e., the tiny fragments with no clear size definition decomposed from large plastic debris by the physical, chemical and biological reactions) [4].

After the attention of microplastics, their sources [5], the harm to the environment [6], the detection methods [7], the regularities of distribution [8], the influence factors [9] and other characteristics have been widely studied. However, these studies have mainly focused on local regions (like some sections/reaches of rivers), lakes, reservoirs, estuaries or sea areas. Although increased studies were reported in recent years, research attention on microplastics’ migration and transformation processes and mechanism in large-scale regions of inland water systems are relatively less. Indeed, migration and transformation are the basis to study the environmental impact of contaminants. The migration of pollutants in the environment is often accompanied with morphological transformation. The migration and transformation of microplastics in freshwater systems is successive. Because of the interconnected relationship between water systems, a study of microplastics in some single river/lake cannot represent the migration and transformation characteristics of this kind of novel pollutant in the aquatic environment.

A marine environment would be the final destination for microplastics from a freshwater system. However, the rest of the contamination might still remain and accumulate in freshwater [10]. During the process of migrating via water flow, microplastics will face various challenges which might prevent their entry into the ocean. Since microplastics have already been presented in human’s daily life, even in our bodies [11], it is urgent to understand their behavior of migration and transformation in freshwater systems, which will help to better realize the migration path and ultimate location and formulate the corresponding management countermeasures. Indeed, there are large numbers of publications including review papers on microplastics published in recent years. However, most of the review papers are focused on the coast and marine environment [12,13]. Not many review papers are focused on the inland water systems, such as rivers, lakes, estuaries and reservoirs. This review expounds the existing form of microplastics in inland water by analyzing their shape characteristics. Furthermore, the migration and transformation characteristics of microplastics in inland water bodies and their influencing factors are reviewed. Finally, the further perspectives of microplastics research are presented. It is expected that this review can help in further studies of microplastics in inland water bodies.

## 2. Status of Current Research

**Figure 1:**
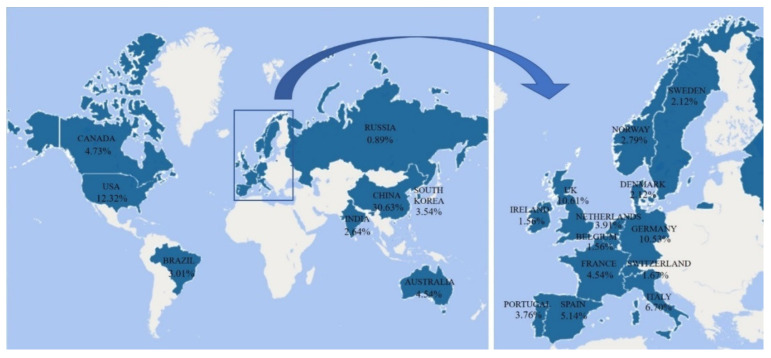
Distribution of microplastics studies at different countries around the world (based on Web of Science searching).

According to the statistics from the core collections of ‘Web of Science’, using ‘microplastic’ and ‘environment’ as subject terms, the searching results returned 2687 papers published from 2008 to 2020 around the world. Among these studies, China contributed 30.63% of the total papers (Figure 1), while the USA and Germany contributed 12.32% and 10.53% of the total papers, respectively.

**Figure 2:**
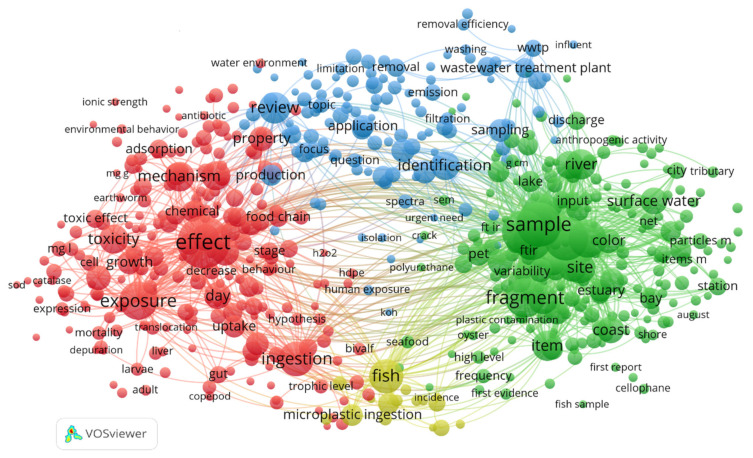
Co-citation analysis of published papers about microplastics around the world.

By further analysis of the 2687 papers using the VOS viewer, the four major clusters (by color in Figure 2) of the microplastics research can be identified (Figure 2). They are impact on the environment, the source of pollutant, distribution characteristics and biological effect. The circles in Figure 2 represent high frequency keyword nodes, while the size of each node reflects the occurrence frequency of keywords. The larger node indicates the higher occurrence frequency and the more important role in the whole network.

## 3. Source of Microplastics in Inland Water Systems

Generally, both the primary microplastics and the secondary microplastics can be eventually discharged into the water body and then migrate into other water bodies accompanying chemical, physical and biological processes.

### 3.1. Primary Microplastics

Primary microplastics have a widespread application in the fields of daily life (such as cosmetic, lotions and toothpaste) [14], industrial manufacture (such as air-blasting media for stripping the metallic surface of airplanes) [15] and medical science (such as drug delivery) [16]. Synthetic fibers made from nylon, acrylic and rayon for the textiles industry can also be recognized as primary microplastics [17]. It has been well noted that these primary microplastics can be discharged into the urban sewage system especially via daily washing products. They will then enter wastewater treatment plants (WWTPs) [18]. However, this organized emission process merely appeared in urban regions. In most undeveloped areas, wastewater is directly discharged into rivers with little or no treatment [19]. Thus, microplastics would easily access freshwater systems without any interception. Although it was found that WWTPs could remove a large amount of microplastics, there was still a large amount of microplastics in effluent due to the vast quantity of daily treated wastewater. These microparticles were then released into the receiving water bodies [20]. In the freshwater system, spherule microplastics are presented at a low abundance, indicating that microplastics in inland rivers are not from primary sources [21]. Yet, even if a small amount of microplastics per liter was released, it can lead to a significant quantity of microplastics intruding into the environment owing to a large volume of sewage [22].

Furthermore, mop pools and washing machines are usually set up in balconies, where the drainage system is connected to the stormwater system. Therefore, wastewater from laundry and household cleaning flows directly into the storm sewage pipes and mostly discharges into the water bodies [21]. Moreover, microplastics removed from sewage are retained in the sewage sludge [23]. Thereafter, in the application of the landfill for the sludge disposal, microplastics can be transferred into the terrestrial environment [24]. Following a series of effects of land/soil transformation, microplastics in soils may finally be introduced into water bodies [25].

### 3.2. Secondary Microplastics

Due to improper disposal and incorrect management, a large quantity of plastic waste enters the environment without treatment and causes a series of environmental pollution problems [26,27]. Once these waste plastics reach the inland water, they would be broken down by hydraulic function while floating up and down in the stream. Meanwhile, mismanaged waste plastics exposed in the environment can be decomposed to smaller sizes by solar ultraviolet radiation, weathering, mechanical abrasion and biodegradation [28]. Under these external forces, plastics lose their structural integrity and get smaller until reaching the microplastic scale [4].

The secondary microplastics originate from fishing nets, films, industrial resin pellets, landfill leachate and other degradable waste plastics [11]. An example is plastic mulching, which is widely used in agriculture to keep land temperature and retain soil moisture for better plant growth. Plastic mulching is a significant source of microplastics in the terrestrial environment, not only in China, but also in other places in the world [29,30]. Once decomposed through weathering, microplastics fallen off from plastic mulching are often reserved in soils [31]. Zhang and Liu [32] found microplastics in soil samples from Yunnan in China, and the concentration is far higher than that found in water. Another example is polypropylene random copolymer (PPR) and the framework materials of an ecological floating bed [33]. They are easily degraded under long-term exposure of ultraviolet radiation and become embrittled especially in the environment below 5 °C. Thus, microplastics can peel off from this perforated plate and directly enter the river. Industrial resin pellets have the same decomposition process. Tyres and road wear particles, identified as secondary microplastics, also have a high contribution of microplastic pollution [34]. According to the statistics, the emission amount of tyre and wear particles for cars, light commercial vehicles and commercial vehicles in China is 0.033 g/km, 0.051 g/km and 0.178 g/km, respectively [35]. As rainwater scours the road in which these particles were deposited, the lighter ones would end up in surface water [36]. In addition, landfill leachate is considered as the high strength wastewater, containing abundant organic matters and microparticles [37,38]. In rural areas of China, household refuse is always piled up at random. These wastes disposed around water bodies will produce leachate containing microplastics and accordingly enter the nearby water bodies.

Additionally, nanoplastics degrading from microplastics have the size of 1~100 nm in diameter [39,40]. The industrial application of nanoplastics is another vital source of plastic particles in the environment [41]. It has been shown that nanoplastics will bring greater risks to nature [4] because they can enter the food chain of aquatic organisms [42], and then might eventually gain access into the human body [43].

### 3.3. Abundance of Microplastics in Inland Water

The average abundance of microplastics in different water bodies varied greatly from almost none to several million pieces per cubic meter. The microplastics detected in China’s inland water systems are summarized in Table 1. Geographically, microplastics pollution has been studied in the Pearl River system, Qinghai inner flow area, Tibet inner flow area, Yangtze River system and Yellow River system, etc. Studies carried out in most areas which have been mentioned investigated two conventional indicators: surface water and sediment. In addition, the other two studied focused on the microplastics in biological samples like fish and Asian clams that have been separately monitored in Qinghai Lake (in north China) and Taihu Lake (in south China). Such different microplastics samples studied can reveal the abundance of microplastics more clearly and integrally. Furthermore, many factors have been studied and explained to influence the quantity of microplastics presented in the inland water environment. These significantly different results from such key factors of sampling sites, sampling methods, inherent natural conditions and physical forces including human population density near the water body, proximity to urban centers, size of the water body and water residence time provide useful input on microplastics transformation [44]. Additionally, if based on the abundance of microplastics alone, microplastics appear to be relatively harmless because those associated data have discrepancy between field-collected microplastics and the microplastics used in the toxicological studies [21]. Therefore, the abundance values are only for reference and further study is needed to perform risk assessments.

## 4. Migration of Microplastics in Inland Water Systems

### 4.1. Migration Paths

Once microplastics are generated from various human activities and enter the water body, their migrating and returning are concerns regarding their fate and the establishment of curbing strategies.

Naturally, microplastics flowing from upstream migrate along the river. Before reaching the ocean, they face the possibilities of predators (aquatic organisms that ingest microplastics by mistake) and colonists (microorganisms that live on microplastics). During these processes, the microplastics might end up in inland water systems.

It is stated that microplastics in an open dynamic freshwater system could eventually arrive in a marine environment [59]. Yet, this might happen in estuaries to ocean or rivers in offshore areas. As transitional zones between rivers and ocean, and human colonies, estuaries play an important role in microplastic migration [60]. More importantly, microplastics found in the ocean might originate from delta urban agglomeration, possessing a high level of economic development and urbanized aggregation. In China, studies have shown that in Jiaojiang, Oujiang, Minjiang and Pearl River estuaries, microplastic pollution is relative to economic structure [61].

In the upstream of a river, the treated effluent of WWTPs carrying residual microplastics was discharged into the river. Some of the microplastics may remain in the river due to their characteristics or surrounding circumstances [53]. Others may continue to flow due to the hydrodynamic conditions [62]. Then, the rest of the particles flowed downstream until reaching the water source intake of drinking water treatment plants (DWTPs) in the downstream city. During the process of microplastics migration, DWTPs are one of the probable destinations for these tiny materials [63].

With the existence of microplastics in freshwater, drinking water safety deserves further consideration. The surface water is the main source water for DWTPs to produce tap water, which means there is a probable risk of microplastics in drinking water. A study conducted by Pivokonsky et al. [64] has shown that both freshwater and drinking water contain microplastics in Czech Republic DWTPs. The same situation was also found in China recently [65,66]. Zhang et al. [65] sampled tap water from seven residential districts of Qingdao city and the analysis results showed a higher abundance of microplastics in tap water than that in source water. Tong et al. [66] investigated 38 tap water samples from different cities in China, while the abundances of microplastics are from 0 to 1247 particles per liter. Although the concentration of microplastics in drinking water is relatively minor, the impact of this quantity cannot be neglected. Tap water might be a significant source of microplastics in human bodies.

Furthermore, in inland water bodies, especially remote mountain lakes, microplastics have to travel a long way to gain their final entry into the ocean. The amount of microplastics arriving in the ocean from the inland water system has not been precisely quantified. The source of microplastics entering the ocean is not specified, whether originating from inland rivers or not. A recent study conducted by Panno et al. [67] showed that two karst aquifers in Illinois, USA, have found microplastics with a concentration of nearly 16 particles per liter. This finding of microplastics in groundwater provides a showcase of the hydrological connections between groundwater and surface water. This might be a possible destination for microplastics in inland enclosed water systems. The inland rivers that cannot flow into the ocean will converge into inland lakes or eventually disappear. Microplastics in these inland water systems might infiltrate into groundwater or alternatively return to urban water-supply systems via groundwater exploitation. This makes the loop of the “migration” and transformation of microplastics further complicated.

### 4.2. Returning

**Figure 3:**
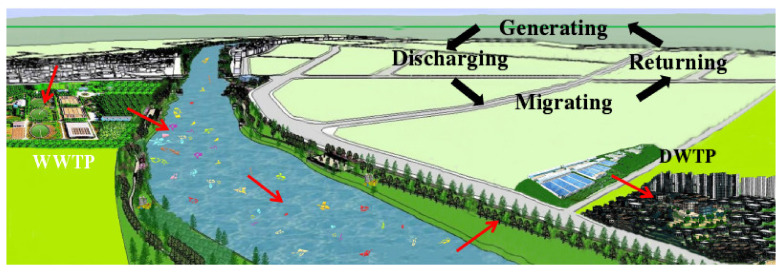
The migration process of microplastics in inland river.

It has been evidenced that if microplastics appear in the tap water, humans would eventually ingest them, leading to microplastic accumulation in human bodies [67]. Meanwhile, aquatic organisms may consume microplastics by mistake [68]. As the food chain continues to humans, those who are the ultimate consumers of the aquatic biota will accumulate microplastics in their bodies [69]. Thus, the fate loop of the microplastics migration becomes clear, i.e., generating from human society, discharging into freshwater systems, migrating in water bodies, reaching a marine environment and eventually returning back to human society, as illustrated in Figure 3.

### 4.3. Influence Factors

The “migration” of microplastics in inland water systems is a very complex process with many influencing factors. Firstly, the migration of microplastics is greatly influenced by hydraulic conditions in the freshwater system including the velocity and depth of the river. Low flow rates and deeper depth can cause microplastics to deposit. On the contrary, high flow rates and shallow depth can cause the deposited microplastics to move [70]. It is shown that the deposition of microplastics occurs in a low energy environment. During storm events, microplastics can be transported by rapid flow and deposited to the deeper position when turbulence slows down [62]. Through the analysis of the water and sediment samples in Wei River in Northwest China, Ding et al. [53] found that the sediment samples had a higher abundance of microplastics than in the water samples, which demonstrated that the lower water velocity and high sand concentration could lead to microplastic accumulation and affect their distribution profile. Secondly, the size of the microplastics is another important factor that cannot be ignored. Studies have shown that micrometer-sized particles are easier to deposit than nanoplastics and microplastics in rivers, thus microplastics and nanoplastics could transport preferentially downstream [71]. It could explain the lack of millimeter-sized particles in the marine environment according to the study of Cózar et al. [72]. Thirdly, seasonal variation may affect the deposition of microplastics. Nel et al. [73] found that the sedimentation of microplastics in winter (160.1 ± 139.5 particles per kilogram) was higher than that in summer (6.3 ± 4.3 particles per kilogram), which may be attributed to the low river flow during winter (0.01–0.57 m/s), allowing for easier deposition. Fourthly, rainfall is another influencing factor of microplastic “migration” in inland water systems. A recent study of Lake Donghu in China found that the concentration of microplastics from the lake in the initial period of rainfall (29.6 items/L) was higher than that in the later period (7.4 items/L) [74]. There are still numerous influence factors affecting the “migration” of microplastics in inland water systems, which remain to be further studied.

## 5. Transformation of Microplastics in Inland Water Systems

The transformation of microplastics in inland water systems is poorly understood. Generally, the bulk of microplastics could be divided into debris of varying sizes. Debris then goes to different directions in certain water systems.

### 5.1. Fate of Microplastics in Water Systems

In rivers, debris can be resolved deeply, precipitated in the sediment or ingested by aquatic animals. Those still in the water would eventually end up in the ocean through open and dynamic rivers or remain and accumulate in isolated and static rivers. It is worth noting that not all the pieces of plastics coming from Yangtze River watershed, for example, can be transferred to the sea. They also have the possibility of depositing at the bottom of the riverbed [55]. However, to the closed water systems like the rivers from Tibet Plateau and Indian Rivers, the microplastics tend to drain into isolated lakes which cause the adverse effects [75].

In lakes or reservoirs, the decrease of flow velocity promotes the precipitation of debris that is heavier than water [21]. As Xiong et al. [47] showed, Qinghai Lake is a sink for the accumulation of microplastics which can gradually break down into small pieces so as to accelerate its deposition. Dam interception makes reservoirs suitable for the accumulation of debris as well. Zhang et al. [51] demonstrated that reservoirs can be the underlying and significant areas for the accumulation of debris. For instance, a high abundance of microplastics was observed in samples collected from the Three Gorges Reservoir (TGR) of China [50]. Moreover, the abundance of microplastics displayed an increasing trend as close to the dam.

**Figure 4:**
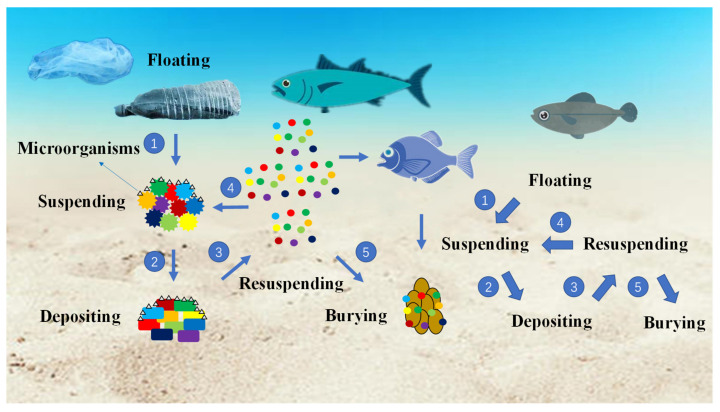
The transformation of microplastics in water column.

The transformation of microplastics in the water body requires five stages of their fate, as shown in Figure 4. To begin with floating plastics, these wastes experience the transformation of morphology by hydrologic function to a more suitable size for floating or depositing. This process is specific to the secondary microplastics. The suspending microplastics can either be taken mistakenly by aquatic animals or be colonized by microorganisms, then deposited in the riverbed under the action of gravity. For the deposited microplastics, on the one hand, they will resuspend from the bottom by the hydrology effects (e.g., flooding). On the other hand, they will permanently deposit at the bottom of the river/lake. Additionally, the resuspended microplastics will continue the same path of suspending, depositing or burying like the aforementioned particles. This creates a transformation cycle of microplastics in a freshwater system of floating, suspending, depositing and burying.

### 5.2. Mechanism of Transformation

#### 5.2.1. Characteristics

Microplastics are different from the traditional environmental pollutants. They can induce many synergistic effects, depending on their properties such as compositions, sizes, densities and shapes [76]. Microplastics can be floated, suspended or sunk in water related to those characteristics.

Microplastics can generally be classified into six shapes. They are fiber, film, fragment, sheet, spherule and foam. Fiber microplastics were found to be the highest abundance, especially in the organism [32]. The two polymer materials, polypropylene (PP) and polyethylene (PE) microplastics, are the majority of textures in conventional microplastics, which have been detected over years. Microplastics can also be detected to consist of cellophane (CP), polyethylene terephthalate (PET), rayon and polyester (PES) in inland water systems [32]. Different areas of microplastics vary in sizes, like estuaries, urban waters, lakes, etc. However, the most considerable property is their density to determine the location in which microplastics exist in water columns. The density most possibly affects the transformation process of the microplastics [48].

#### 5.2.2. Processes

Spatially, the process of the vertical transformation of microplastics in water bodies is rather complicated as microplastics are found in water bodies, columns, surfaces and shores [77]. Lower density microplastics exist in the water surface, while high-density particles can sink to the lower location [16,78]. For example, microbeads (part of primary microplastics) remain suspended in the water column or floating in the water surface due to low density and small size [62,79]. However, most of the microplastics can be transferred downstream along the current to the marine environment or the closed lakes. The rest is deposited in the sediment by degradation. In addition, microplastics are transported to the organism bodies via indirect or direct uptakes. Aquatic organisms such as fish mainly ingest microplastics by predation actions [80]. These activities from the water body or sediment pose potential risks to animals at high levels in a trophic chain [81]. Microplastics can enter human bodies by chronic accumulation if people eat fish which contain microplastics.

#### 5.2.3. Degradations

Degradation means that microplastics have been exposed to various conditions for a long time, leading to their morphology and characteristics being altered to a certain extent [82], including photodegradation, mechanical degradation, thermal degradation and biodegradation [83].

Most debris is susceptible to oxidative degradation induced by UV and visible light in terms of photodegradation [84]. Microplastics can be subjected to photodegradation once floating on the water surface in inland water systems, hence photo-oxidative degradation is regarded as a main degradation option once microplastics are exposed to these specific conditions. Mechanical degradation occurs through microplastics’ exposure to mechanical actions such as stirring, blowing and shaking at any stage, whereas it particularly happens greatly after chemical and biological degradation [85]. Some studies stated that the mechanical degradation of microplastics in inland water systems has greatly to do with hydraulic conditions and other compositions [32]. Thermal degradation through high temperature and low humidity can make structures of microplastics loosened and flexible so as to break down into fragments.

Biodegradation is a relatively slow biochemical transformation of microplastics by biota, which can change the properties and textures on the surface initially by microorganisms’ attachment and occupation [86]. Microorganisms are likely to colonize and aggregate on the surface of microplastics, which will probably change the collective buoyancy and lead to an increasing density [87]. As such, the floating debris can provide a removable habitat for microorganisms to form a microbiota community, either sinking with increasing density or being food for aquatic organisms [46]. Microplastics ingested by microorganisms will transform into the deeper position of a water column via defecation or predation by benthic animals [50]. Cole et al. [88] indicated that zooplankton fecal pellets play a vital role in transporting microplastics to deeper waters and can facilitate the movement of contaminants throughout its vertical flux. In addition, microplastics, depositing on the bottom, are likely to be reentered to the water column by erosion or resuspension of the riverbed. They would then launch a new cycle of movement until being buried in the riverbed permanently [89].

## 6. Effects and Potential Risks of Microplastics in Inland Water Systems

The potential adverse effects of microplastics in inland water systems can be derived from physical, chemical and biological aspects [77,90].

Physical impacts are mainly divided into entanglement and ingestion in water systems. Entanglement is often reported and could pose fatal threats to living species in water bodies. Once entangled, these organisms are prone to drown, suffocate, strangulate or starve [91]. In the event of their predators appearing while they are entangled, they are bound to die. The ingestion of microplastics by aquatic animals can continually accumulate along trophic levels up until reaching human bodies [92]. Laist [93] stated that based on the records related to entanglement and ingestion, marine microplastics are affecting at least 267 individual species worldwide.

The chemical impacts dominate greatly. Microplastics may sequentially contribute to toxicity impacts on human bodies and aquatic organisms through ingesting [77]. These phenomena are due to the fact that microplastics could transfer toxic substances and heavy metals as a carrier [94,95]. The toxins from the microplastics are released to the water systems via degradation consequently gaining entry into the food chains, which would also accumulate in organisms through the bioaccumulation process [83]. For instance, Wardrop et al. [94] conducted a study to show that microbeads from personal care products have the potential to transfer adsorbed pollutants to the rainbow fish and then ingest them. The uptake and the accumulation of a kind of microplastics in zebrafish along with the toxins in livers were investigated and detected by Lu et al. [96].

The biological effects state that it is possible for microorganisms to attach and colonize the targeted microplastics. Thiel and Gutow [97] found that microorganisms such as bacteria can quickly attach onto the surface of microplastics and move to the microplastics which are generally more durable and firm than others. The other biological effect is an alterable physical property due to the ubiquitous presence of biofilms as coatings which occupy the surface of microplastics and they are usually observed to completely encapsulate the microplastics [98]. No doubt, further study is required to systematically and comprehensively analyze the potential hazardous effects of microplastics in the water systems [99]. Overall, the aforementioned factors are required for further study in line with microplastics’ fates and their effects.

## 7. Perspectives and Further Research Needs

By unremitting efforts made in recent years, the characteristics, toxicity, ecological efficiency and movement patterns of microplastics have been gradually investigated. For the moment, it has been well accepted that, regarding the main route of the microplastics in inland water systems, the various morphology changes of microplastics originated from the plateau, then flowed downstream to the river and eventually ended up retaining the lake or the ocean.

Indeed, correlation studies on migration and transformation patterns of microplastics in the major river systems are not fully understood. In inland rivers, the migration path of microplastics derived from terrestrial sources is still unclear, while the interconversion relationship of microplastics in the terrestrial environment and aquatic environment is unclear as well. Although a large part of existing studies indicated the occurrence of microplastics and the abundance, it is very necessary to analyze the source of microplastic pollution due to the complicated ways of accepting pollution in inland water systems. At present, studies on the abundance of microplastics in each process of the migration closed circle (generating, discharging, migrating and returning) should be carried out. This will provide technical support and a theoretical basis for determining the source and route of microplastic pollution in inland water systems.

In addition, although there are studies on the distribution of microplastics at estuaries, they are still rarely on the movement mechanism of microplastics. For the complexity of the estuary environment, the questions, like how do microplastics migrate and transform at this special site, and how seriously do microplastics affect the estuary environment, remain unaddressed. Therefore, the future studies should focus on determining the amount of microplastics originating from a remote inland river, confirming the influence of hydrodynamic parameters and seasonal variation on the transportation of microplastics from inland river to the sea.

**Figure 5:**
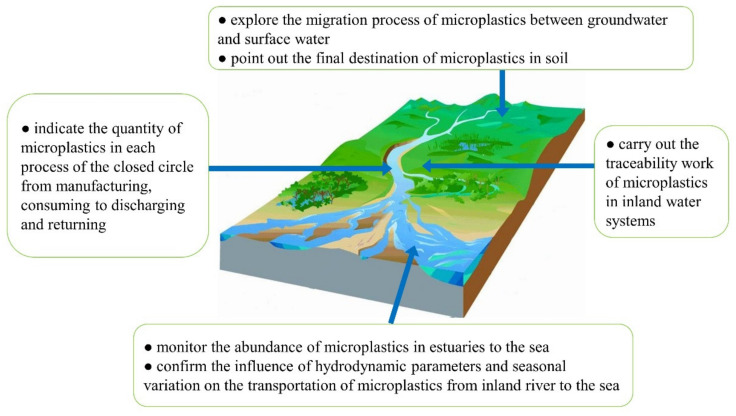
Suggestions for microplastics study in inland water systems.

On the other hand, further studies are highly desirable to develop the control strategy and explore the removal mechanism of the microplastics. According to the current status, some suggestions for further studies of microplastics in inland water systems are proposed and illustrated in Figure 5.

Relatively, the migration process of microplastics in closed water systems is not very clear. Obviously, this requires the studies to focus on the movement mechanism of microplastics in such kinds of lakes/rivers. In addition, the migration ways and behavior of microplastics in riverbeds and underneath aquifers should be jointly studied. As one of the important water sources, the microplastic pollution extent of groundwater should be taken into account. Accordingly, corresponding control strategies of microplastics in groundwater can then be formulated.

It is important to mention that the monitoring technique of microplastics, especially the national or international standard procedure, should be established. This is vital for microplastic study, allowing the comparison of different results across the world. Last but not least, it should be pointed out that the policies of plastic use and responsibility of plastic pollution play a key role in microplastics control. Public awareness of plastic pollution, plastics manufacturers and policy makers are all responsible for the issue of microplastics. It is believed wiser that the integrated actions should be applied to significantly reduce the source of microplastics and then promote their curbing in the water environment.

## 8. Conclusions

Microplastics have attracted international attention in recent years. Based on the updated literature, this review has identified:

(1) Microplastics in the marine environment are the resultant of the migration and transformation processes/behavior of microplastics in inland water systems. The migration and transformation processes could form a closed and complicated circle by food chain.
(2) As for the migration, the microplastics’ fate is from manufacturing, consuming, discarding to migrating and returning to the human society. For the transformation, microplastics experience five stages of their fate in an inland water system. These include changing into suspending pieces; ending up deposited as the sediment; resuspending under various changing conditions; ending up via burying into the soil as the part of the riverbed; reaching the marine environment; and being ingested by organisms and also becoming entangled with aquatic plants.
(3) The migration and transformation processes/behavior of microplastics could be affected by not only the carrying flow of the river itself, but also the vertical condition (depth) of the water body.
(4) Future studies should focus not only on determining the amount of microplastics, but also on confirming the influence factors of the inherent natural conditions of hydrodynamic parameters. It is reasonable to understand that each inland water system has its own geographical and geological feature, but integrated actions should be taken to explore the role of the human population density near the water body, plastics manufacturers and public awareness of plastic pollution on the effects of microplastics’ migration and transformation processes/behavior.

## Figures and Tables

**Table 1:** Studies on detecting microplastics in inland water systems.

Water Body	References	Collected Sample	Mean Abundance (Sample Position)
Beijiang River (in Feilaixia)	[45]	Surface water & Sediment	0.56 ± 0.45 items/m^3^ (surface water)
Pearl River	[46]	Surface water & Sediment	2724 items/m^3^ (surface water)
Qinghai Lake	[47]	Surface water &	180,900 items/km^2^ (surface water)
		Sediment & Fish	364 items/m^2^ (sediment)
			5.4 items per individual (fish)
Rivers and lakes in Tibet plateau	[48]	Surface water & Sediment	132 items/m^2^ (surface water)
Taihu Lake	[49]	Surface water &	3.4–25.8 items/L (surface water)
		Sediment & Asian clams	11.0–234.6 items/kg dw (sediment)
			0.2–12.5 items/g ww (Asian clams)
Three Gorges Dam (TGD)	[50]	Surface water & Sediment	1597–12,611 n/m^3^ (surface water)
	[51]		25–300 n/kg ww (sediment)
Urban water areas in Changsha	[52]	Sediment	270.17–866.59 items/kg
Wei River	[53]	Surface water & Sediment	3.67–10.7 items/L (surface water)
			360–1320 items/kg (sediment)
Yangtze River Estuary	[54]	Surface water & Sediment	4.92 × 105 items/km^2^ (surface water)
	[55,56]		34 items/kg (sediment)
Charleston Harbor, USA	[57]	Surface water	413.8 ± 76.7/m^2^
Bostanu, Persian Gulf	[58]	Surface water	1258 ± 291/kg

## Data Availability

Not applicable.
